# A Compact Immunoassay Platform Based on a Multicapillary Glass Plate

**DOI:** 10.3390/s140509132

**Published:** 2014-05-23

**Authors:** Shuhua Xue, Hulie Zeng, Jianmin Yang, Hizuru Nakajima, Katsumi Uchiyama

**Affiliations:** Department of Applied Chemistry, Graduate School of Urban Environmental Sciences, Tokyo Metropolitan University, 1-1 Minamiohsawa, Hachioji, Tokyo 192-0397, Japan; E-Mails: xue-shuhua@ed.tmu.ac.jp (S.X.); zeng-hulie@tmu.ac.jp (H.Z.); yang-jianmin@ed.tmu.ac.jp (J.Y.); uchiyama-katsumi@tmu.ac.jp (K.U.)

**Keywords:** multicapillary glass plate, compact immunoassay, enzyme-linked immunosorbent assay

## Abstract

A highly sensitive, rapid immunoassay performed in the multi-channels of a micro-well array consisting of a multicapillary glass plate (MCP) and a polydimethylsiloxane (PDMS) slide is described. The micro-dimensions and large surface area of the MCP permitted the diffusion distance to be decreased and the reaction efficiency to be increased. To confirm the concept of the method, human immunoglobulin A (h-IgA) was measured using both the proposed immunoassay system and the traditional 96-well plate method. The proposed method resulted in a 1/5-fold decrease of immunoassay time, and a 1/56-fold cut in reagent consumption with a 0.05 ng/mL of limit of detection (LOD) for IgA. The method was also applied to saliva samples obtained from healthy volunteers. The results correlated well to those obtained by the 96-well plate method. The method has the potential for use in disease diagnostic or on-site immunoassays.

## 1. Introduction

The development of simple, rapid immunoassay approaches is of tremendous importance for on-site diagnostics and for monitoring highly infectious diseases [1]. Infectious diseases, such as the avian influenza virus, severe acute respiratory syndrome (SARS), hand-foot-and-mouth disease, influenza H1N1 and H7N9 are all threats to human health/life and collectively cause losses to social/economic development [2,3]. Therefore, developing methods for the rapid on-site assay of these diseases at an early stage is extremely important. The enzyme-linked immunosorbent assay (ELISA) is one of the most widely used approaches in clinical diagnostics, food safety testing, and environmental monitoring [4,5]. The technique is usually carried out in a 96-well microtiter plate and involves a series of tedious processes, including sample introduction, incubation and washing. Moreover, substantial amounts of sample are consumed and the method is time-consuming and sometimes suffers from low sensitivity [6]. In order to overcome these drawbacks, various efforts have been devoted to developing new methods for diagnostic immunoassays. For example, ELISAs have been integrated with a micro/nano system to increase the surface/volume ratio and improve reaction kinetics [7–9]. A variety of materials, such as quantum dots [10], photonic crystals [11], membranes [12] or papers [13], have also been used in conjunction with immunoassays.

On the other hand, the microfluidic technique, with its advantages of miniaturization and integration has also been widely exploited for use in immunoassays [14]. However, in the microfluidic-based immunoassay, a single microchannel is always used as the working space for an immunoassay [15] and this has the limitation of a small working surface. Similarly, a glass plate or glass capillary, which have also been exploited for use in immunoassays [16,17] also have limitations in that a flat surface or a single channel is used. Interestingly, a MCP with uniformly paralleled microchannels would be an ideal candidate for use in an immunoassay. Such a material has been used for DNA hybridization reactions [18], in which MCP showed substantial advantages over flat glass, in terms of its higher sensitivity and a faster hybridization rates. We previously reported on the use of MCP combined with a microbead-based chemiluminescence immunoassay [19], in which it simply acted as a switch for bonding/free protein separation. However, the use of the inner surface of the MCP, which can increase the immobilization capacity of an antibody to improve the reaction efficiency of an immunoassay, does not appear to have been utilized.

We report herein on the development of a compact immunoassay utilizing microchannels within the MCP, which has a large surface area and high surface-to-volume. Thus, the MCP ratio could not only increase coating capacity, but also accelerate an assay. The method would be expected to decrease overall immunoassay time because of the short diffusion distance in the microchannels of the MCP. The compact immunoassay system shows considerable potential in terms of speed and high-sensitivity for on-site diagnosis and for rapidly monitoring a disease.

## 2. Experimental Section

### 2.1. Reagents and Apparatus

A human IgA kit, including affinity purified goat anti-human IgA (1st Ab), a human reference serum solution and goat anti-human IgA HRP conjugated (2nd Ab), was obtained from Bethyl Laboratories (Montgomery, TX, USA). Bovine serum albumin (BSA) was obtained from Merck (Calbiochem, Darmstadt, Germany). 10-Acetyl-3,7-dihydroxyphenoxazine (Amplex® Red reagent) was purchased from Life Technologies (Invitrogen, Eugene, OR, USA). It was dissolved in dimethylsulfoxide (DMSO) to a concentration of 13.8 mmol/L for use as a storage solution and was stored in refrigerator at −20 °C prior to use. The working substrate solution of Amplex® Red was prepared by mixing with phosphate-buffered saline (PBS) buffer (pH = 7.4) and H_2_O_2_ solution just before use. Na_2_HPO_4_, NaH_2_PO_4_ and H_2_O_2_ were purchased from Wako Pure Chemical Industries Ltd. (Osaka, Japan). Washing buffer (PBST) was prepared with 0.5% (w/v) BSA/0.1% (v/v) Tween-20 (Kanto Chemical Co., Inc., Tokyo, Japan) in 100 mmol/L PBS (pH = 7.4). 10 mmol/L NaOH (Kanto Chemical Co., Inc.) and 10 mmol/L HCl (Wako Pure Chemical Industries Ltd.) were prepared for the cleaning of the MCP. All solutions and buffers were prepared with deionized water purified by Milli-Q system from Nihon Millipore (Tokyo, Japan). All buffers used in the experiments were filtered through 0.45 μm membrane filter (Jhwpo 4700, Nihon Millipore). A neutral detergent (TCN-5) used for plate cleaning was obtained from Tomisc Company (Tokyo, Japan)

A polydimethylsiloxane (PDMS) kit (Silpot 184) was obtained from Dow Corning Toray (Tokyo, Japan). MCP (1 mm in thickness, effective area 20 × 20 mm, capillary inner diameter 20 μm) was obtained from the Incom Company (Charlton, MA, USA). A Branson 5210 ultrasonic cleaner was obtained from Yamato Industry (Tokyo, Japan). A 96-well plate reader (SpectraFluor Plus) was obtained from Tecan (Melbourne, Australia). An Espec ST-110 high temperature oven (Osaka, Japan) was used for the PDMS thermo-curing. An incubator (SN-M 40S) was obtained from the Nissin Co. (Yokohama, Japan).

### 2.2. Setup

**Figure 1.**
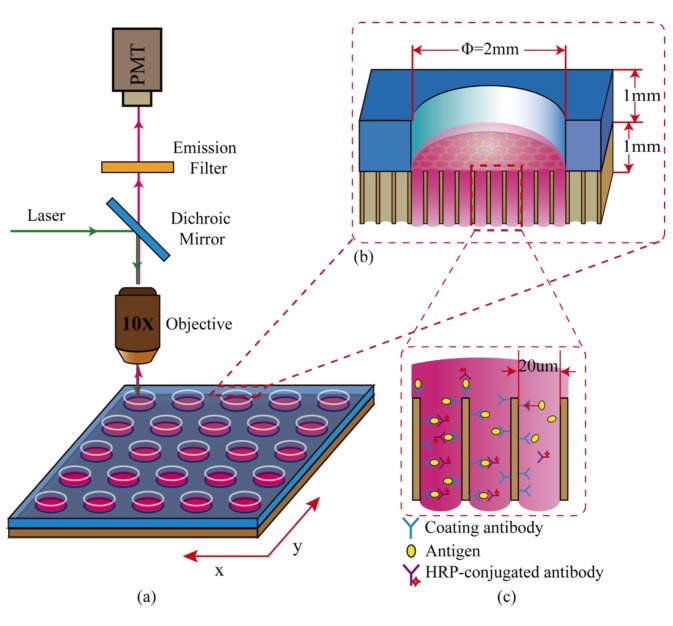
Schematic diagram of the compact immunoassay in a microwell array on MCP, integrated with an x-y stage and a laser-induced fluorescence measurement system (**a**), cross-section of a single microwell consisting of MCP and a PDMS slide (**b**), and the schematical illustration of the compact ELISA performed in microchannels within MCP, in which each microchannel with 20 μm diameter is acted as a micro-reactor with short diffusion distance (**c**).

Figure 1a schematically illustrates the setup used for the microwell device and the system construction. The microwell immune device was composed of a MCP and a PDMS slide. The PDMS slide with a 5 × 5 hole-array (Φ = 2 mm for each hole) was reversibly attached to the surface of the MCP to produce a 5 × 5 well array. The device was placed onto a laboratory-made holder combined with an electromotive x-y stage for movement to the detection point. Measurements were carried out by an incident light fluorescence microscope (BX-51, Olympus, Tokyo, Japan) system equipped with filter set XF-102 (Opto Science, Inc., Tokyo, Japan) integrated with a 543.5 nm laser (3478EC, Melles Griot, Carlsbad, CA, USA). The fluorescence signal was detected by a photomultiplier (PMT) (H7422-40, Hamamatsu Photonics, Hamamatsu, Japan), and the signal was amplified by means of a laboratory-made current amplifier, the magnified analog signal was then converted to digital data by a 12-bit A/D converter (PicoScope 4224, Pico Technology, Cambridgeshire, UK) and recorded with a PC (Dell Inc., DCNE, Beijing, China). Figure 1b shows a cross-sectional view of a microwell consisting of the MCP and PDMS slide. The antibody/antigen recognition reaction took place in the compact microchannels in the MCP, and each microchannel acts as a micro reactor, as shown in Figure 1c.

### 2.3. Fabrication of the Multicapillary Glass Plate Assembled Microdevice

A PDMS slide was prepared by mixing the PDMS prepolymer and curing crosslinker at a mass ratio of 18:1 and the resulting mixture was degassed *in vacuo*. The mixture was then poured onto a cleaned glass, which was fenced with Scotch tape, followed by curing at 65 °C for 1 h. The PDMS slide was sequentially peeled off and drilled with a punch (2 mm diameter) for each well to form a 5 × 5 microarray (20 mm × 20 mm). The PDMS slide was immersed into a 50% ethanol solution and then deionized water for ultrasonic cleaning for 30 min, respectively. After cleaning, it was dried at 80 °C for 10 min. At the same time, the MCP was successively cleaned with a neutral detergent, deionized (DI) water, 0.01 mol/L HCl, DI water, 0.01 mol/L NaOH, DI water. It was then dried at 80 °C for 20 min. Finally, the cleaned MCP and the above cleaned PDMS slide were attached to form the microarray (as shown in Figure 1), and the attachment was reinforced by heating at 80 °C for 10 min.

### 2.4. Performance of ELISA Method

#### 2.4.1. Assay Protocol for ELISA Using the Microarray Device

A sandwich immunoassay was performed to detect h-IgA to confirm the concept of the developed compact immunoassay. A coating solution of the 1st Ab was pipetted into MCP-constructed microwells at an amount of 2.0 μL/well, followed by incubation in a humidity controlled cabinet (RH = 97 ± 5%) at 37 °C for 30 min. After incubation, waste solutions were removed by touching the bottom of the MCP with a filter paper (No.51B, Advantec, Tokyo, Japan). Then, 3.0 μL/well of PBST buffer was introduced into the microwells for washing, and the waste solutions were also moved away by touching the bottom of the MCP with a filter paper. The washing procedures were performed three times. A BSA (1%, w/v) solution was next introduced into each microwell with 2.5 μL/well for blocking. After 30 min incubation in a 97% RH cabinet at 37 °C and 3 washings with PBST buffer with 3.0 μL/well, different concentrations of h-IgA solutions were introduced into each microwell.

After a 10 min incubation in the 97% RH cabinet at 37 °C, each microwell was washed 3 times with PBST buffer. The 2nd Ab solution was then introduced into microwells at a volume of 2 μL/well. After a 10 min incubation, the unabsorbed antibodies were removed by washing five times with PBST buffer. Finally, 2.5 μL/well of an Amplex® Red substrate solution (6.9 mmol/L Amplex® Red, 50 mmol/L H_2_O_2_, 100 mmol/L PBS) was pipetted into each well to generate Resorufin for fluorescence detection.

#### 2.4.2. Assay Protocol for ELISA Using the 96-Well Plate

The h-IgA measurement was also performed on a 96-well plate following the traditional ELISA protocol. Briefly, 1st Ab solution (10 μg/mL) was initially pipetted into a 96-well plate at 100 μL/well, and incubated for coating at 4 °C overnight. After washing three times with 200 μL/well of PBST buffer, a BSA (1%, w/v) blocking buffer was introduced at 200 μL/well and incubated for 1 h at room temperature. After the plate was washed three times with PBST (200 μL/well), different concentrations of h-IgA solution were introduced into the plate at 100 μL/well and the plate was incubated for 1 h at room temperature. Each concentration of h-IgA was performed with three replicates. The plates were then washed with PBST three times, and 100 μL of the 2nd Ab solution (2.5 μg/mL) was added to each well. After 1 h of incubation at room temperature, the plates were washed with PBST. Finally, Amplex® Red substrate was added at 100 μL/well to generate Resorufin for fluorescence detection.

#### 2.4.3. Saliva Sample Measurement

Saliva samples were collected from six healthy volunteers at around 10:30 in the morning and were placed in polypropylene tubes (15 mL). The samples were centrifuged at 10,000 rpm for 30 min. The supernatant was then isolated, and diluted with PBS (0.01 mol/L, pH 7.4) buffer. Before the ELISA, samples with different dilution factors ranging from 10- to 10,000-fold were prepared in PBS buffer and tested. The measurements of h-IgA in saliva were carried out using the microwell array following the above ELISA procedures. At the same time, the saliva samples were also measured by the 96-well plate method using the same dilutions as the reference.

## 3. Results and Discussion

### 3.1. Optimization of Detection Point

**Figure 2.**
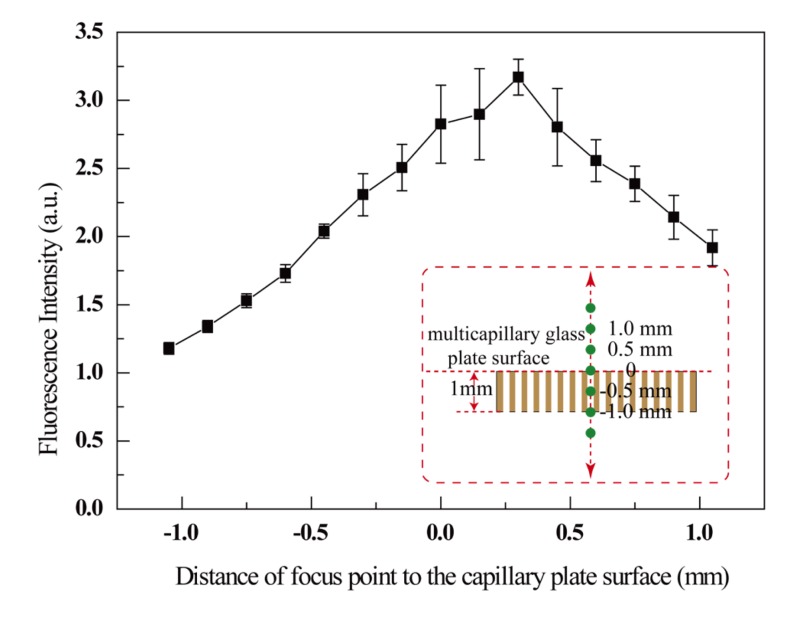
The effect of laser focusing point for fluorescence detection in the microwell was investigated by measuring a Resorufin solution (50 μmol/L). The inserted schema illustrated focusing point moving up and down vertically to the surface of the MCP.

Laser induced fluorescence (LIF) measurement is one of the most sensitive detection techniques for an immunoassay. However, optimization of the detection point in the LIF system is extremely important for obtaining reproducible signal recording. Consequently, Resorufin which is the product of the reaction of Amplex® Red with H_2_O_2_ catalyzed by HRP was used to investigate the effects of the detection point in the microwell. As shown in Figure 2, focusing the point of the laser onto the capillary surface was easily accomplished using the microscope. When the focus point was moved up/down or away from the MCP surface, the signal response changed accordingly. From the results, a slight lifting of the focus point resulted in an improved fluorescence recording for both signal intensity and reproducibility due to the increase in the excited surface area.

### 3.2. MCP-Based Immunoassay

In this study, MCP with uniform microchannels (20 μm inner diameter, 50% open ratio) was utilized as the solid matrix for immobilizing the coating antibodies (1st Ab) in ELISA. Since the amount of immobilized 1st Ab was the critical parameter that affected the capability of catching the target antigen, this is critical in terms of the reaction efficiency of an immunoassay. Additionally, the diffusion controlled rate of the reaction was also decreased greatly due to the small size of the microchannels. As summarized in Table 1, the valid surface area of the proposed method for the immobilization of coating antibody was about four times larger than that of a traditional 96-well plate. Moreover, the surface-to-volume ratio was greatly increased, which accelerated the antibody-antigen reaction with minimal reagent consumption. Therefore, the MCP-based microfluidic chamber array can be used to perform ELISA measurements and is highly efficient compared to competing methods.

The other benefit of the microchannel in MCP was the shortened diffusion distance for the interaction of the antibody and antigen [20]. As shown in Table 1, the diffusion length of the antigen (from the center to the inner surface of the microchannel) was shortened from 3.5 mm to 0.010 mm for the traditional 96-well plate and the MCP, respectively. This results in a tremendous decrease in the diffusion time for the antigen to contact the antibody on the surface. Meanwhile, due to the small size of the microchannels, the injected liquid entered in the channels driven by capillary force [21]. While with the liquid moving into the channel and travelling down to the end tip of the microchannels, the upward capillary force holds the water pillar inside the microchannels [22]. Therefore, the solution could be held in a stable orientation to permit incubation in ELISA. On the other hand, the solution could be easily removed by touching a filter paper to the bottom of the MCP, which disrupted the balance between capillary force and gravity force and allowed the liquid to pass through the microchannels. By such a performance with liquids stop/flow, the washing in ELISA was made extremely simple.

### 3.3. Optimization of Some Conditions for h-IgA Measurement

**Figure 3.**
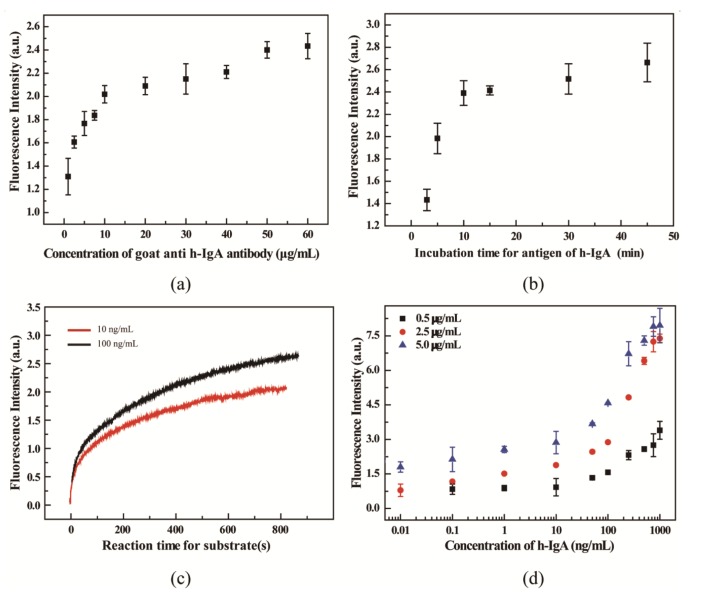
Investigation of optimum conditions for ELISA on a microwell array on MCP. (**a**) Effect of the concentration of coating Ab on ELISA. h-IgA: 0.2 ng/well, incubation time: 30 min under 97% RH, 37 °C; (**b**) Influence of incubation time on ELISA performance. Coating Ab: 10 μg/mL, h-IgA: 0.2 ng/well; (**c**) Kinetic curves of the Amplex® Red substrate reaction with the H_2_O_2_ catalyzed by 2nd Ab. Coating Ab: 10 μg/mL, h-IgA: 0.02 ng/well. (**d**) Effect of the concentration of 2nd Ab on the measurement of h-IgA in microwell array on MCP.

As shown in Figure 3a, the concentrations of the coated 1st Ab were investigated in the range of 2–60 μg/mL, while the h-IgA concentration was fixed at the 100 ng/mL. As shown in the figure, the fluorescence intensity of the signal increased dramatically up to a concentration of coated 1st Ab of 10 μg/mL. We chose 10 μg/mL of the 1st Ab for the designed microassay. Figure 3b shows the influence of incubation time in the microwell immunoassay. The optimal incubation time was determined to be 10 min. As a result, the incubation time was greatly decreased from hours to minutes compared with that for the 96-well plate shown in Table 1. The reason for this can be attributed to the shorter diffusion distance of the microchannel inside the MCP. To investigate the speed of the enzyme catalytic reaction and determine the optimal detection time, plots of the enzyme reaction kinetics of the substrate solution were recorded as shown in Figure 3c. The fluorescence signal increased rapidly up to a time of 200 s after the substrate introduction. Thus, the measurements were performed at 220 s in the following experiments. Additionally, the effect of the 2nd Ab was also investigated, in an attempt to further enhance the efficiency of enzymatic reaction and to decrease the background. As shown in Figure 3d, 0.5, 2.5 and 5 μg/mL of the 2nd Ab solutions were investigated, respectively. The data showed that a lower concentration of 2nd Ab resulted in lower signal intensity and a poor correlation. To the contrary, the higher concentration of the 2nd Ab generated a higher background signal and relative standard deviation (RSD). Therefore, 2.5 μg/mL of 2nd Ab was selected as the optimal concentration considering the background and RSD.

### 3.4. Investigation the Effects of Blocking

The specificity and sensitivity of the assay can be influenced by the non-specific adsorption (NSA) of other proteins or biomolecules by occupying the active sites of the valid surface. Actually, various strategies have been employed to reduce NSA, in attempts to decrease the background signal, and enhance the sensitivity of an immunoassay. A simple strategy, involving surface blocking, is usually adopted to reduce the NSA. Therefore, we investigated the blocking conditions. Experimentally, bovine serum albumin (BSA), casein, and SuperBlock buffer were used to search for the best blocking option.

**Figure 4.**
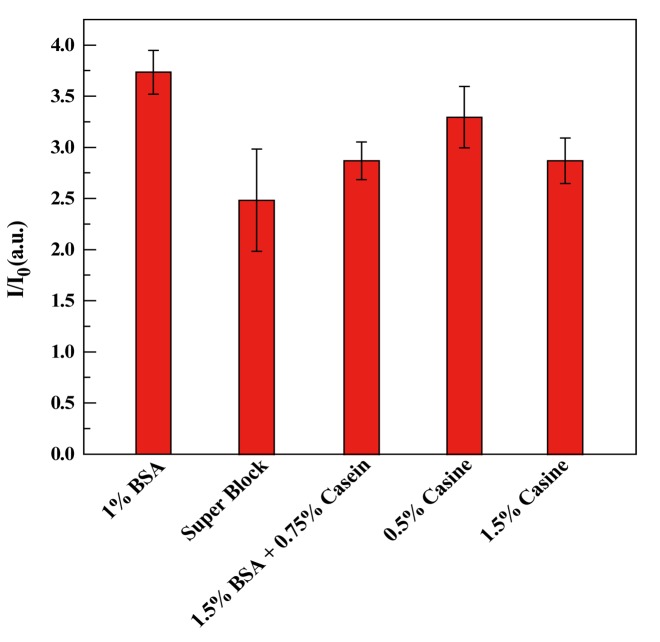
Effect of blocking solutions on the ELISA performed in a microwell array on MCP. The signal to noise ratio (S/N) was calculated from the fluorescence intensity of 100 ng/mL h-IgA solution (S) divided by the blank signal (N).

Figure 4 illustrates the blocking efficiencies of the above blocking solutions. The BSA solution obviously showed best blocking efficiency. Thus, a 1% BSA solution was the most efficient, and was adopted in the following experiments.

### 3.5. Investigation the Influence of Substrate Concentration and the Calibration of h-IgA

**Figure 5.**
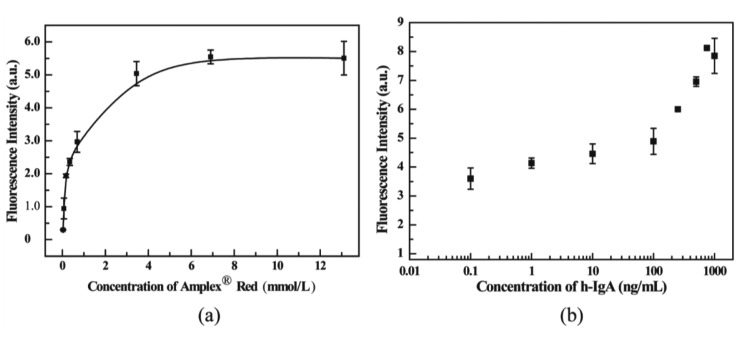
Effects of the substrate concentrations on the enzyme catalytic reaction performed in the present method, the other conditions were used with the optimal ones as the former works investigated (**a**). Plot for h-IgA detected by the present method under the optimized conditions (**b**).

In order to obtain the optimum sensitivity in an immunoassay, the reaction between Amplex® Red and H_2_O_2_ catalyzed by the HRP-conjugated antibody (*i.e.*, 2nd Ab) was investigated. As shown in Figure 5a, the signal intensity was increased with increasing concentration of substrate in the enzyme reaction, while for the present method, the optimum concentration of the substrate was selected as 6.9 mmol/L, since the curve reached a plateau at that concentration. This ensured that the quantitative measurement of ELISA in the microwell array was possible in the present immune approach. Therefore, h-IgA was measured by the present microwell immunoassay under optimal conditions. From the results shown in Figure 5b, the assay was a success and showed a high sensitivity. Because of the microsize of the well, sample consumption was cut to 2 μL/well, thus the absolute amount of target could be dramatically decreased. Meanwhile, the LOD was calculated to be 0.05 ng/mL by the average signal of the blank ( $\bar{{\text{S}}_{\text{blank}}}$) plus three times the standard deviation of the blank (3SD) [6,23]. The contradistinctive data for h-IgA measurements by the microwell array and the 96-well plate method are listed in Table 2. The method showed a better LOD than the traditional 96-well plate method. Regarding reagent consumption, the immunoassay time and the size of the device, the proposed MCP-based microwell was superior to those for the traditional 96-well plate.

### 3.6. Comparing with 96-well Plate in the Measurement of Saliva

Saliva is an important bodily fluid, and samples can be obtained easily and noninvasively. Moreover, it contains various substances and biomarkers that can be used as indicators of health and disease status [24,25]. To evaluate the applicability of the present method in a practical ELISA, h-IgA in saliva samples were measured, and it was also measured by the 96-well plate method as a reference. In this experiment, the saliva samples were collected from six healthy adults. The resulting samples were diluted with PBS buffer, and assayed.

**Figure 6.**
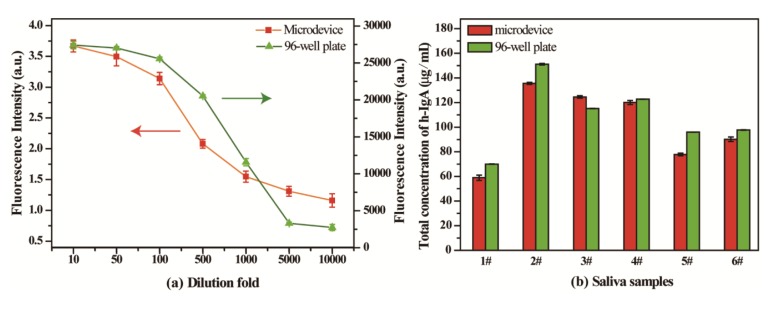
Measurements of saliva sediments by the present microwell and 96-well plate method: Investigations of the dilution factor on the measurement of h-IgA (**a**); Detection concentrations of h-IgA in saliva samples with 500-fold dilutions (**b**).

Since problems associated with matrix effects and working range are frequently encountered in measurements of biological samples in immunoassays, we investigated the dilution factors in the measurements of h-IgA by the proposed microwell approach as well as the traditional 96-well plate method. Based on different sample dilutions, the findings show that the microwell method showed a good relative analytical sensitivity and a good dilution tolerance in h-IgA measurements (see Figure 6). In addition, h-IgA in six saliva samples at a 500-fold dilution were tested by the two methods and the results were highly correlated (r^2^ = 0.91). In addition, when recovery trials by adding standard h-IgA were carried out by the proposed method, the obtained recovery ratio was in the scope of 93.7%–112.2%, as listed in Table 3.

## 4. Conclusions/Outlook

The design of a novel compact immunoassay using a MCP constructed microwell array is described. The enlarged surface area and decreased microsize of the condensed micro channels in the MCP resulted in a rapid and efficient antibody-antigen recognition. Additionally, by taking advantage of the large surface area-to-volume ratio and a microwell array, the performance of the immunoassay was fast, involved minimal sample consumption as well as high-density measurements. This permits high-throughput detection to be performed on a 20-mm square MCP of simple construction with a high degree of sensitivity and a short assay time. The developed immunoassay method was successfully used in the measurement of h-IgA and the results were in agreement with results obtained using the traditional 96-well plate method. The use of the method for analyzing saliva samples suggests that the method has potential for use in on-site diagnostics. For further enhancement of the detection sensitivity, a large amount of sample could be accepted because of the high surface area of this method. Thus the proposed method could be greatly improved by using an on-line concentration method, which is now being pursued in our laboratory.

## Figures and Tables

**Table 1.** The physical parameters of microwell array and 96-well plate.

**Parameter**	**Microwell**	**96 Well Plate**
Diameter of well (mm)	2	7
Valid surface area (mm^2^)	314	76.93
Volume (μL)	1.57	100
Surface-to-volume (ratio)	200	0.7693
Diffusion distance from center (mm)	0.01	3.5
Diffusion time (s)	0.5	6.125 × 10^4^

**Table 2.** Comparison of the ELISA performance in the present microwell array and 96-well plate.

		**Microwell**	**96-Well Plate**
Reagent consumption (μL)	Goat anti-h IgA solution	2	100
	Blocking buffer	2.5	200
	Antigen/sediment solution	2	100
	HRP-conjugated goat anti-h IgA solution	2	100
	Substrate solution	2.5	100
	Washing buffer	42	2400
Total reagents consumption (mL)		0.053	3
Assay time (min)		25	135
LOD (ng/mL)		0.05	5.19

**Table 3.** Investigation of recoveries for h-IgA in saliva samples by the proposed microwell array.

**Sample**	**h-IgA Addition (ng/mL)**	**Detection (ng/mL)**	**Recovery**
1	0	190.83	---
	25	214.25	93.7%
	50	242.25	102.8%

2	0	218.83	---
	25	242.65	95.28%
	50	274.93	112.2%
